# Patient-derived organoids: a promising tool for breast cancer research

**DOI:** 10.3389/fonc.2024.1350935

**Published:** 2024-01-26

**Authors:** Yixin Shi, Zhanwen Guan, Gengxi Cai, Yichu Nie, Chuling Zhang, Wei Luo, Jia Liu

**Affiliations:** ^1^Liaoning Laboratory of Cancer Genomics and Department of Cell Biology, College of Basic Medical Sciences, Dalian Medical University, Dalian, China; ^2^Translational Medicine Research Institute, First People’s Hospital of Foshan, Foshan, China; ^3^Department of Breast Surgery, The First People’s Hospital of Foshan, Foshan, China

**Keywords:** breast cancer, patient-derived organoids, tumor microenvironment, drug screening, individualized therapy

## Abstract

Breast cancer (BC) is the most prevalent malignancy among women worldwide. Traditional research models such as primary cancer cell and patient-derived tumor xenografts (PDTXs) have limitations. Cancer cells lack a tumor microenvironment (TME) and genetic diversity, whereas PDTXs are expensive and have a time-consuming preparation protocol. Therefore, alternative research models are warranted. Patient-derived organoids (PDOs) are a promising in vitro model. They mimic the TME, gene expression, and cell types of original cancer tissues. PDOs have been successfully developed from various cancers, including BC. In this review, we focused on the value and limitations of PDOs in BC research, including their characteristics and potential in drug development, personalized therapy, immunotherapy, and the application prospects of PDOs in drug testing and prognosis.

## 1 Introduction

Breast cancer (BC) is a prevalent malignancy among women globally. In 2020, BC surpassed lung cancer to become the most frequently diagnosed malignancy (1). Rapid advances in drug testing and high-throughput sequencing technology in the past few decades have resulted in considerable progress in the early-stage screening and clinical treatment of BC (2). Approximately 70%–80% of early-stage BCs can be cured with surgery and targeted therapy, with the mortality rate of patients with BC decreasing each year. However, approximately 685,000 patients still succumb to late-stage BC each year; therefore, it remains the leading reason for mortality among women worldwide (3).

At present, two-dimensional (2D) tumor cell lines and patient-derived tumor xenografts (PDTXs) remain the commonly used cancer research models (4). Although these experimental models have played a substantial role in basic BC research in the past, limitations remain.

**Figure 1:**
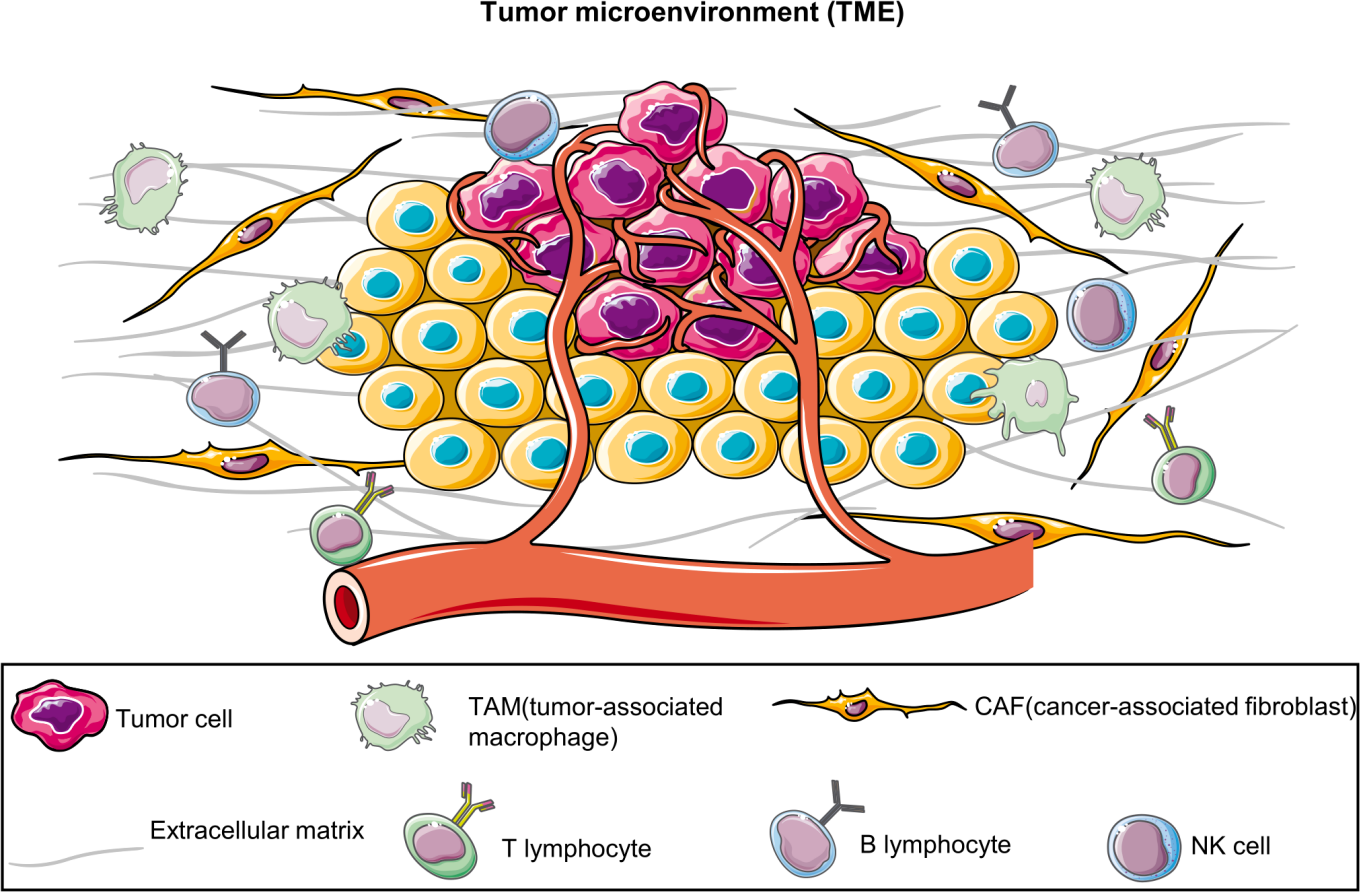
Tumor microenvironment (TME). TME comprises diverse cell types, including tumor cells, immune cells, vascular endothelial cells, fibroblasts, and the extracellular matrix. They collectively interact to affect tumor growth, metastasis, and treatment responses. The establishment of patient-derived organoids can effectively preserve these components.

BC emergence and progression involve processes other than tumor cell proliferation; they are intricately associated with the tumor microenvironment (TME) (5, 6), including tumor-associated fibroblasts (7), tumor-associated macrophages (8), adipocytes (9, 10), immune cells (11, 12) and the extracellular matrix (ECM) (13) (**Figure 1**). However, 2D-cultured tumor cells lack not only the TME but also the genetic heterogeneity of the original tumor owing to the substantial genetic changes that occur in multiple clones (14). Furthermore, although PDTXs can preserve the TME and the complex tumor structure, they also have many limitations, including higher experimental costs, longer experimental time, increased technical requirements, low success rate, and ethical issues (4). Therefore, alternative models for BC research are warranted.

In 2009, Sato et al. illustrated for the first time that a solitary leucine-rich repetitive G-protein-coupled receptor 5 (LGR5)-positive intestinal stem cell can form three-dimensional (3D) epithelial structures. These structures exhibit distinct polarized epithelial characteristics, featuring proliferative crevices and differentiated villous sections, and can maintain stable growth in vitro (15). Subsequently, this culture technique has facilitated the formation of other kinds of epithelial organoids, and patient-derived organoid (PDO) models have been developed from different organs and cancer types (4), including the liver (16), breast (17), colon (18), lungs (19), and stomach (20). Recently, 3D culture technology has emerged as a promising in vitro model for cancer. Different from monolayer cells and PDTXs, PDOs are 3D cultures that are formed in vitro from surgically resected or biopsy tissues, which are structurally and functionally similar to in vivo organs. They not only provide a good summary of the gene expression profiles, personalized characteristics, and cell types of native tissues but also preserve the TME of a patient’s tumor. In addition, organoids possess self-organization and self-renewal properties (14, 21, 22).

## 2 Overview of organoid development in BC

**Figure 2:**
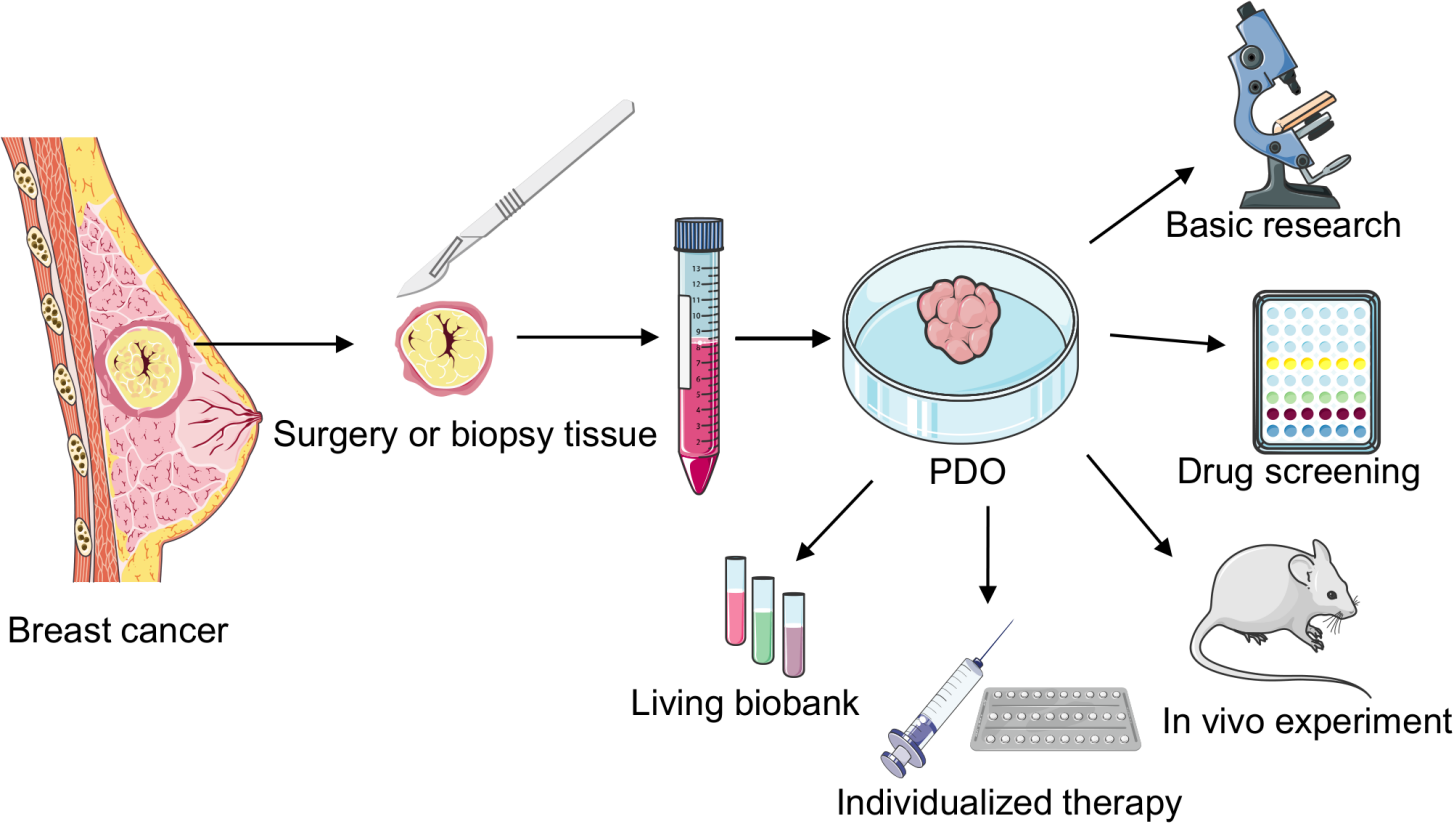
Cultivation and application of breast cancer (BC) patient-derived organoids (PDOs). PDOs derived from surgically resected or biopsied tumor tissues undergo enzymatic digestion to generate intricate organoid structures. These organoids are cultured after centrifugation, finding applications in diverse areas such as fundamental research, rapid drug screening, biobank development, in vivo transplantation studies, and individualized clinical treatments.

In 1979, Emerman et al. cultured normal mammary epithelial cells in collagen gel and observed that different from the 2D culture model, the collagen gel matrix allowed the entry of nutrients to the basolateral cell surface, cells to approach the medium surface and gas phase, the interaction of epithelial cells with matrix elements, and sublaminar flexibility, allowing changes in cell shape. This approach provided a unique microenvironment for breast epithelial cells in a 3D environment (23). In 1982, Bissell et al. studied BC organoids for the first time and noted that the presence of the ECM in 3D models can better simulate gene expression in BC cells (24). This study not only demonstrated the importance of the TME but also shifted the understanding of BC cells from 2D to 3D. Furthermore, in 1982, M Hiratsuka et al. isolated and cultured breast organoids with duct-like structures from breast biopsy tissues (25). In 1990, a study revealed the varying effects of distinct ECM compositions on murine mammary tissue-derived cells (26). In 1992, Bissell reported for the first time that normal primary mammary epithelial cells and biopsied cancer cells could be cultured to generalize the growth behavior and structural and functional differentiation of these cells in vivo (27). In 2007, Bissell et al. revealed that vital cellular signals disappeared when cells were cultured outside their natural environment on flat 2D plastic surfaces. However, a significant portion of these essential microenvironmental cues were reinstated using 3D laminin-rich extracellular matrix cultures (28). Since Sato et al. first cultured intestinal organoids from a single intestinal epithelial stem cell in 2009, BC stem cells have also gradually gained importance as the key to organoid formation (17). In 2012, breast Lgr5+ cells not only served as breast stem cells and survived for a long time in the passage process but also developed normal ductal structures similar to the breast via cytokine arrangement (29). Sachs et al. cultured 95 BC organoids from 155 surgical BC tissues and successfully established the pioneering BC organoid repository; they observed that the histopathology and hormone receptor status of BC organoids were similar to those of the original tumor; furthermore, they noted that BC organoids can be used for in vitro drug screening and respond similarly as in vivo xenografts and patients (19). Recently, with the continuous improvement and optimization of experimental techniques, the success rate of BC organoid culture has reached 87.5% (30). Therefore, BC organoid technology is widely employed in basic research, drug screening, and individualized therapy (**Figure 2**).

## 3 Advantages of PDO models

PDO models exhibit various captivating advantages, which transcend those offered by traditional cell and patient-derived xenograft (PDX) models (4, 14, 19).

### 3.1 Advantages of PDO over cell models

1. Simulated TME: Using the 3D model, the interactions between tumor cells and epithelial cells, immune cells, and fibroblasts, all of which are present in the TME but absent in 2D cell lines, can be studied. Therefore, PDOs more accurately replicate the in vivo TME, including cell–cell interactions, the ECM, growth factors, and cell signaling pathways. Furthermore, they more precisely reflect the biological characteristics of tumors, making it a research platform that resembles biological reality (31).
2. Preservation of heterogeneity: Heterogeneity is gradually lost during 2D cell culture; however, the TME remains highly heterogeneous. This suggests that the 2D culture microenvironment is less heterogeneous than the original TME. Compared with 2D models, PDOs exhibit higher heterogeneity (32).
3. Individualized treatment: Tumor-derived cell lines cannot fundamentally interact with the original tumor, which provides stromal support to cancer cells in the form of fibroblast elements, blood vessels, and immune mediators. For example, in a previous study, only seven prostate cancer cell lines were generated, with only four expressing androgen receptors (33). Furthermore, only a few cell lines have been generated for glioblastoma; these established glioma cell lines do not represent primary human gliomas (34). Therefore, cell lines cannot be a model to study personalized treatments. However, because PDOs originate from patient tissues, they provide robust support for individualized treatment, helping in formulating optimal treatment strategies based on the pathological characteristics and gene expression patterns of patients.
4. Biological complexity: PDOs can simulate more intricate biological processes, including cell–cell interactions and signal transduction, helping researchers gain deeper insights into the mechanisms underlying tumor development.

### 3.2 Advantages of PDO over PDX models

1. No need for animal experiments: Animal experiments are not needed to establish PDO; therefore, ethical and legal concerns related to animals and potential adaptational changes from interspecies transplantation are avoided.
2. Convenient sample acquisition: The collection of patient samples is relatively straightforward; in contrast, the establishment of PDX models requires intricate animal experiments and sample processing procedures.
3. Long-term cultivation: PDO can be maintained for an extended period in vitro, facilitating the long-term observation and evaluation of treatment effects.

## 4 BC PDOs recapitulate the heterogeneity of original tissues

Tumor heterogeneity refers to the concept that tumor cells can exhibit distinct genetic and phenotypic characteristics, including gene mutation, cell morphology, and gene, protein, and marker expression. Furthermore, because of genetic and phenotypic differences, tumor cells exhibit differences in proliferation, transfer ability, and drug resistance. Tumor heterogeneity can be categorized into two types: inter- and intratumor heterogeneity. Intertumor heterogeneity refers to the diversity observed across distinct tissues. For example, tumor occurrence and development are different in each patient; furthermore, the treatment process is different, which leads to tumor heterogeneity among different patients. On the other hand, intratumor heterogeneity primarily depends on the TME and intrinsic characteristics of tumor cells (35). Compared with monolayer cultures, PDOs can better summarize the heterogeneity of original tumor tissues. Diermeier et al. performed RNA sequencing (RNA-seq) to compare the tumor and organoid transcriptome profiles of luminal B (MMTV-PyMT) and HER2/nue-amplified (MMTV-Neu-NDL) mouse models. They observed that, in most instances, the organoids could effectively replicate the expression profile of the initial tumor tissue. Only a small fraction, approximately 1% of the gene transcriptome (142 of 13,854 expressed genes), exhibited statistically significant differences between the tumor and day 6 organoids. The affected genes were predominantly protein-coding genes (85%), affecting their connected signaling and metabolic pathways. Simultaneously, noncoding RNA levels were largely unaltered (36). Sachs et al. successfully created 95 organoid biobanks from patients’ tumors, representing all BC subtypes. Then, they compared the whole genome sequences of organoids and primary BC tissues via whole genome sequencing and RNA-seq and observed that the transcriptomic, histological, and genomic fingerprints of the original tumor tissue were largely unchanged in the organoids after 20 passages. Furthermore, they performed H&E staining to histopathologically analyze tumor tissues and organoid sections and observed that the phenotype of BC organoids was often similar to that of the original BC tissue. For example, solid organoids are produced for ductal carcinoma, whereas loose organoids are produced for lobular carcinoma. In addition, nuclear atypia is preserved in organoids, including enlarged and polymorphic nuclei and high mitotic activity (19). In the context of the BC model, preserving the key and common specific biomarkers, including estrogen receptor (ER), progesterone receptor (PR), and human epidermal growth factor receptor-2 (HER2), is vital. These biomarkers serve as vital targets for drugs against BC (37). Chen et al. successfully established organoids from 132 patients with BC and evaluated the status of ER, PR, HER2, and Ki-67 on organoids and tumors via immunohistochemistry. They observed that the expression patterns of these BC markers were well preserved, regardless of whether the organoids had received systemic anticancer therapy (38). Bhatia et al. generated an organoid bank from 83 BC tissues. They not only elucidated that the organoids accurately replicated the traits of the patient’s tumor but also observed that when triple-negative breast cancer (TNBC) organoids were implanted into NOD/SCID mice, the resultant tumors were morphologically similar to the original tumor (39). In a recent study, Saeki et al. developed PDOs from 10 patients with BC and observed that these BC PDOs had different growth rates and patterns. Subsequently, they subjected these PDOs to transcriptome analysis at the single-cell level, innovating novel analytical techniques: (1) clustering cells within each PDO, (2) discerning genes specifically expressed in each cluster, and (3) quantifying their resemblance to subsequently categorize the clusters. These analyses revealed that each PDO comprises multiple subpopulations with different cell states and cycles, estrogenic responses, and epithelial–mesenchymal transition-like gene expression programs. This suggests that PDO-specific cell clusters reflect different PDO properties. Moreover, they subjected two other BC tissues or PDO pairs to RNA-seq and revealed that although a complete one-to-one correspondence was not achieved, PDOs retained some degree of tumor heterogeneity of the original tissue after successive passages (40).

## 5 Clinical application of organoids

### 5.1 PDOs as a preclinical BC model for drug discovery

PDOs can be used as a preclinical model for drug testing to elucidate the medicinal value of some compounds or small-molecule antagonists, thereby opening new avenues for developing novel pharmacological treatment modalities for cancer (41). Wu et al. identified that MS023, a type I protein arginine methyltransferase (PRMT) inhibitor that is presently in clinical development and not yet approved for use, exhibits antitumor growth activity in TNBC cell lines. However, they observed that TNBC PDOs exhibited different drug responses to MS023, with only 50% of TNBC PDOs being sensitive to this inhibitor and the remaining PDOs exhibiting resistance. Furthermore, they noted that the TNBC cells most sensitive to MS023 treatment exhibited PRMT1, which may be a therapeutic target for MS230. This study provides important insights into the pharmacological mechanisms underlying MS230 to develop targeted therapies for TNBC and other cancers and potential novel opportunities for cancer immunotherapy (42). Moreover, Sun et al. revealed an association between elevated SOST expression and bone metastasis in BC; this correlated with decreased survival rates among patients. Interestingly, SOST inhibition markedly decreased the bone metastatic capacity of SCP2 cells. Mechanically, SOST interacts with STAT3 to amplify the TGF-β/KRAS signaling pathway, thereby promoting BC growth and facilitating bone metastasis. To explore novel agents for patients with BC and bone metastasis, Sun et al. screened ~120,000 compounds from a small-molecule chemical library and observed that compound S6 significantly inhibited SCP2 cell proliferation in a dose- and time-dependent manner and effectively inhibited BC PDO growth. As a result, they identified S6 as a candidate compound for further development as a novel cancer therapeutic agent (43). Ma et al. performed a high-throughput screening of large tumor suppressor (LATS) kinase inhibitors using an in vitro LATS kinase assay and identified one effective LATS inhibitor, i.e., VT02956, from ~17,000 compounds. They observed that VT02956 inhibits ESR1 expression by targeting the Hippo pathway, resulting in the growth of ER^+^ BC cells and patient-derived tumor organoids, with little cytotoxicity in other cells. They concluded that LATS is an unexpected target for cancer therapy, particularly for endocrine-resistant breast cancer, and provided a novel idea for treating ER^+^ BC (44). JNK-IN-8 is a c-Jun N-terminal kinase (PRMTs) inhibitor that plays a tumor suppressor role in osteosarcoma, colorectal cancer, and pancreatic cancer; however, it has not been used in clinical trials. Soleimani et al. noted the significant inhibitor effect of JNK-IN-8 on TNBC cells, with translucent cytoplasmic vacuoles with lysosomal characteristics. Furthermore, JNK-IN-8 effectively inhibited the growth of TNBC PDOs, exhibiting rupture and disintegration phenotypes. Except for inhibiting JNK, JNK-IN-8 activates lysosomal biogenesis and autophagy by targeting TFEB/TFE3. Their results suggest that JNK-IN-8 is a novel and promising treatment for TNBC in the future (45). BC stem-like cells (BCSCs) are the potential reason for the recurrence, metastasis, and drug resistance of TNBC. In 2023, Liu et al. observed that CRM1 expression was significantly increased in the tumor tissue samples of patients with BC; this was closely associated with a poor prognosis. They developed LFS-1107, a highly effective small-molecule synthetic analog that targets CRM1 in cells. LFS-1107 can inhibit TNBC cells at low concentrations and selectively remove CD44^+^CD24^−^ BCSCs. Furthermore, in a mouse xenotransplantation model, LFS-1107 strongly inhibited tumor growth and removed BCSCs in residual tumor tissues. LFS-1107 also prompted the nuclear retention of survivin, inhibiting the transactivation capabilities of STAT3 and subsequently decreasing the downstream expression of stemness regulators. In addition, Liu et al. cultured PDOs from the tumor tissues of patients with TNBC and observed that LFS-1107 inhibited TNBC PDOs more significantly compared with approved cancer drugs. Finally, they demonstrated that LFS-1107 can enhance the killing effect of chemotherapeutic agents and downregulate multidrug resistance-associated protein targets (46). Although PDO provides valuable tools for drug discovery, challenges in standardization and scalability may limit its widespread applicability.

### 5.2 PDOs can predict the response of patients with BC to drugs

**Figure 3:**
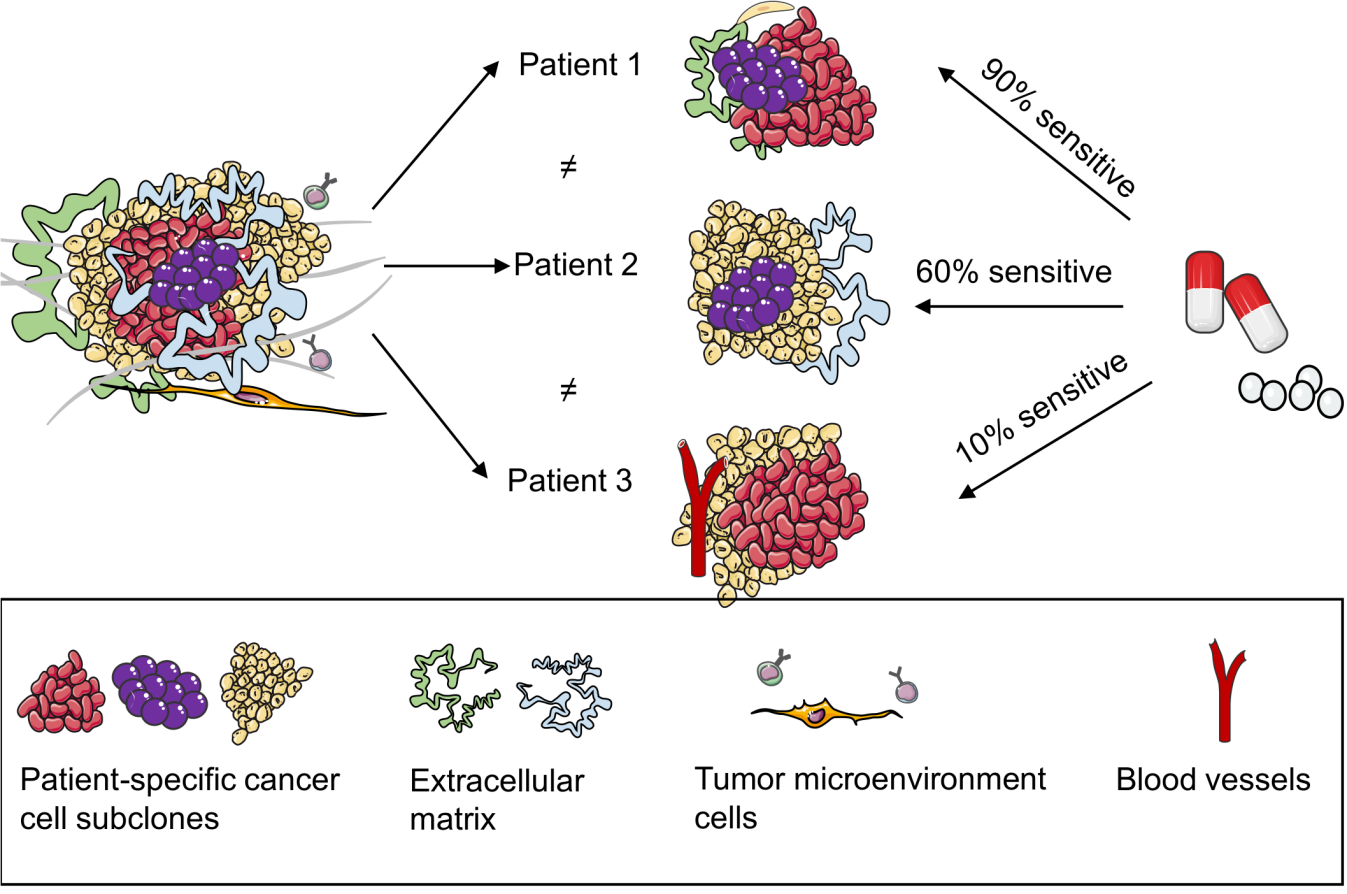
Patient-derived organoids (PDOs) can predict the responses of patients with breast cancer to drugs. PDOs present a model for elucidating the heterogeneity within patient tumors, adept at preserving multiple cell types and subpopulations inherent to the patient’s tumor. Subsequently, they proficiently retain the drug response characteristics of the patient’s tumor, thereby promoting the accuracy of predicting the drug efficacy for individual patients.

To date, several organoids have been cultured from tumors directly sourced from patients and healthy breast tissues, facilitating the establishment of an organoid repository. In addition to capturing the diverse characteristics and histology of a patient’s tumor, these organoids can also predict the responsiveness of an individual patient to specific drugs. Subsequently, they offer insights into the expected drug reactions of patients (19, 47, 48) (**Figure 3**). Shu et al. collected 17 biopsy samples from suspected patients with BC; five organoids were successfully constructed from the tissue biopsies. They tested the sensitivity of the organoids to different neoadjuvant chemotherapy (NAC) drugs and observed that different BC PDOs responded differently to the NAC drugs. For example, BCb20, BCb27, and BCb30 belonged to HR-positive organoids; however, BCb20 was sensitive to epirubicin, and BCb27 and BCb30 were resistant to epirubicin. Furthermore, BCb22 and BCb21 belonged to HER2-positive organoids, whereas BCb22 was sensitive to carboplatin, but BCb21 was resistant to carboplatin. Finally, Su et al. compared the clinical responses of patients with BC with the results of in vitro experiments and observed that the responses of PDOs to each drug were consistent with the clinical responses of patients. Therefore, they concluded that PDOs can be utilized to predict a patient’s response to monotherapy and a patient’s efficacy to a combination of drugs or NAC, eventually leading to individualized treatment. To compare drug responses in both in vitro and in vivo contexts, Sachs et al. generated 12 BC organoids from the needle biopsies of 13 patients diagnosed with metastatic BC. They reported how these patients responded to standard-of-care treatments. For drug susceptibility tests, they selected two BC organoids sensitive and resistant to tamoxifen. The in vitro responses of BC organoids to tamoxifen were consistent with the responses of corresponding patients; this suggests that BC organoids can be utilized as predictive in vitro substitutes for BC in vivo (19). In addition, Guillen et al. observed that the drug responses of PDX-derived organoids (PDxO) are consistent with responses in vivo. They used 16 PDxO models to screen 45 compounds. Thereafter, they selected the drug that exhibited a unique pattern of response or resistance in the PDxO models and tested the drug in vivo on the PDX model. Most PDX models exhibited drug responses similar to the PDO models (49). Collectively, BC PDOs provide a more accurate and promising research model for predicting drug responses in patients with BC, leveraging its ability to simulate patient conditions. However, while the prospects are optimistic, further validation is warranted to ensure reliability. Nevertheless, PDO holds promise in advancing individualized cancer treatment strategies.

### 5.3 TME and immunotherapy

TME refers to a complex network of cells, stroma, and signaling molecules that surround tumor cells and play an essential role in tumor growth, invasion, metastasis, and therapy responses. The fundamental constituents within the TME are as follows (50–52):

1. Incorporating immune components: Immune cells such as T cells, B cells, natural killer cells, macrophages, and dendritic cells have a pivotal effect on the TME. Their interactions significantly shape tumor immune reactions and subsequently affect the efficacy of therapeutic interventions. Stromal cells such as fibroblasts, endothelial cells, and smooth muscle cells constitute the tumor stroma; they provide support for tumor growth and invasion.
2. ECM: The ECM comprises proteins, polysaccharides, and other substances, and it forms the tumor stroma. The ECM plays a vital role in tumor cell migration, invasion, and cellular signaling.
3. Blood vessels: Neovascularization formation plays a vital role in tumor growth and nourishment; this is closely associated with the malignancy and prognosis of tumors.
4. Cytokines and growth factors: Cytokines and growth factors such as tumor necrosis factor-α, interferons (IFNs), and vascular endothelial growth factor regulate immune responses, angiogenesis, and tumor growth.
5. Interstitial fluid: The interstitial fluid is present in the interstitial spaces surrounding the tumor. It contains information on the tumor’s condition, including cellular fragments, DNA, and RNA.
6. Immune suppressive factors: Factors that inhibit immune responses are present in the TME, including immune checkpoint molecules, which help the tumor in evading immune attacks.

These components interact with each other, collectively forming the intricate TME network, which affects tumor behavior and treatment responses. Among them, immune cells and molecules can affect tumor growth, metastasis, invasion, and therapy responses, playing pivotal roles in controlling and inhibiting tumor development (53).

Recently, tumor immunotherapy has gained considerable attention as a prominent approach to cancer treatment. It activates or rallies the patient’s immune system, amplifying antitumor responses in the TME and regulating the elimination of tumor cells (54). However, owing to the complexity of the human immune system and its ineffective reconstruction, the efficacy of in vitro immunotherapy drug models does not match clinical responses in many cases (55). Therefore, there are considerable challenges associated with immune-tumor models, and in vitro models that can be used to verify the efficacy in individuals are urgently warranted in clinical settings.

Organoids provide a new opportunity for tumor immunotherapy. Owing to the precise recapitulation of immune cell and molecular interactions in the TME, PDOs offer a more authentic platform to elucidate the mechanisms underlying immunotherapy. Furthermore, leveraging PDO models derived from specific patients facilitates the prediction of individual responses to diverse immune-based therapeutic approaches. In addition, conducting drug screening within PDOs allows efficacy and toxicity assessment of various immunotherapy agents, thereby offering crucial insights into the development of novel immunotherapies (56, 57). This approach can expedite the drug development process for immunotherapies (**Table 1**).

**Table 1:** Related studies on immunotherapy and the tumor microenvironment of patient-derived organoids (PDOs).

Model	Immune cell/Composition of TME	Methods	Targets	Outcome	References
BC PDOs	CAFs	Microfluidic device	GPNMB	CAFs enhance the invasion of 3D BC cells by regulating the expression of GPNMB.	(58)
Spheroid of TNBC cells	Peripheral blood mononuclear cell (PBMC)	Co-culture	/	Patients’ PBMC showed a wide range of antitumor responses.	(59)
CRC PDOs	Peripheral blood lymphocytes (PBLs)	Co-culture	Tumor-reactive T cells	Co-culturing PBLs with PDOs generated tumor-targeting T cells that specifically killed organ-matched tumors.	(58)
Diverse tumor types PDOs	T cells, B cells, NK cells	Air-liquid interface (ALI)	TILs	ALI-PODs models successfully simulate immune checkpoint blocking (ICBs), activate tumor antigen-specific TILs, and induce tumor cytotoxicity	(60)
TNBC PDOs	Dendritic cells (DCs)	DC vaccine	Tumor-reactive CD8 T cell	Activated cDC1s induced potent antitumor CD8+ T cell response in low immunogenic TNBC PDOs.	(61)

### 5.4 Drug screening and individualized therapy

In the field of oncology, the importance of individualized therapy is increasingly being emphasized, and PDOs have emerged as an innovative disease model, offering remarkable potential and opportunities for individualized treatment strategies. PDOs offer a distinct edge because they are developed from patient-derived tissue samples, facilitating the precise in vitro emulation of the distinct biological traits exhibited by individual tumors. This fidelity in replicating the unique characteristics and disease status of patients facilitates the generation of laboratory models that mimic the primary ailment of a patient. Therefore, they significantly help deepen our understanding of the fundamental nature of diseases and expedite the exploration of potential avenues for treatment. Furthermore, PDOs can be used to predict a patient’s response to specific treatment modalities, helping clinicians devise optimal therapeutic strategies. By evaluating diverse drugs or treatment protocols, we can identify the most effective therapeutic approach at an individual level. This process contributes to the increased treatment success rates while minimizing unnecessary drug exposure and potential side effects, thereby providing robust support for developing and refining individualized treatment strategies (38, 62, 63). With the continuous optimization of the 3D culture system of PDOs, many laboratories can culture organoids in large quantities. Therefore, PDO models are also employed for the high-throughput screening of different anticancer drugs to rapidly and accurately obtain individualized treatment regimens for patients (41). Chen et al. collected 132 BC tissues from 125 patients, including 77 who did not receive anticancer therapy and 55 who did. They developed 99 personalized cancer models (PDOs) from these tissues and established a drug library with 49 anticancer drugs. By screening the PDOs, they identified the most sensitive drugs for each patient, resulting in the successful development of patient-specific treatment strategies (38). More relevant studies on the applications of PDOs in drug screening and individualized therapy are presented in **Table 2**. BC PDOs represent a tailored treatment model to meet specific patient needs. However, there is potential variability in drug efficacy, and rigorous validation is warranted to ensure the reliability of individualized therapeutic strategies. Furthermore, the widespread application of PDOs remains limited. Despite these challenges, the prospect of improving treatment precision with PDOs remains a driving force in advancing individualized medicine.

**Table 2:** Drug screening and individualized therapy.

Tissue	Source of PDOs	Primary/	Drugs	Outcome	References
Advanced BC	Biopsy	Primary and Multiple lung metastases	Doxorubicin Neverolimus Epirubicin	The tumor resisted five treatments, but PDO drug screening and guided medication can effectively regress it.	(38)
TNBC	Biopsy	Liver metastasis	Bortezomib	Bortezomib, previously used for other types of cancer, showed promising results as a BC treatment for the first time.	(38)
Metastasis BC	Biopsy	Primary	Tamoxifen	BC PDOs’ tamoxifen responses mimic patients’ responses, showing potential as in vivo breast cancer surrogates.	(19)
Advanced BC	Biopsy	Primary, Cancerous pleural effusion, and Cancerous ascites	Resveratrol	Resveratrol had a higher efficacy than common anticancer drugs in advanced BC	(64)
TNBC	Biopsy	Primary, Liver metastases and Bone metastases	Eribulin	PDOs can be used to screen the optimal therapeutic drugs for patients, but they cannot comprehensively summarize the systemic diseases of the patients.	(49)

### 5.5 Studies on the mechanisms underlying tumor progression

Compared with traditional cell lines, PDOs have the advantage of preserving tumor heterogeneity and maintaining a higher degree of TME. Therefore, by studying PDOs, researchers can achieve a more comprehensive understanding of the molecular and cellular mechanisms underlying BC development (4, 65). For example, Priscilla et al. established a BC PDO migration model. Using a 3D microfluidic system and computational model, they discovered that during the collective migration of heterogeneous tumor and tumor stromal cell clusters, the leading migrating cells identify and segregate a specific subset that is characterized by the collective migratory potential, notably the keratin-14 and calponin-3-positive subgroups. This process allows the migrating cells to control their protrusion dynamics by locally producing laminin; this emphasizes how these cells guide collective migration by interacting with the microenvironment (66). Moreover, a study revealed that metformin and simvastatin, two metabolism-related drugs, play crucial roles in modulating hypoxic TME and angiogenesis. Furthermore, the combined use of metformin and simvastatin inhibits BC PDO proliferation, promotes apoptosis, alleviates hypoxia, decreases angiogenesis, and enhances vascular normalization (67). **Table 3** summarizes the studies on other PDOs.

**Table 3:** Applications of breast cancer (BC) patent-derived organoids (PDOs) in research.

Model	Applications	Outcome	References
BC	Combination Therapy	The combined use of Metformin and Simvastatin inhibits BC PDO proliferation, promotes apoptosis, alleviates hypoxia, reduces angiogenesis, and enhances vascular normalization.	(67)
ER^+^BC	Combination Therapy and Immunomodulation	ABT-199 induces Rb dephosphorylation and reduces G1/S phase proteins, thereby enhancing the cell cycle arrest induced by fluorouracil/paclitaxel.	(68)
chemotherapy-treated BC	Mechanisms of tumor immunity and drug resistance	NCOR2 inhibits anti-tumor therapy by modulating HDAC3 to suppress gene expression dependent on IRF-1 and the IFN signaling pathway. Altering the interaction between NCOR2 and HDAC3, reducing NCOR2, or impeding its epigenetic activity enhances chemotherapy responsiveness and restores anti-tumor immunity.	(69)
HER2^+^ BC, TNBC	Mechanism of anti-HER2 resistance	The synergistic effect of anti-FGFR4 and anti-HER2 therapies in breast cancer with intrinsic or acquired resistance.	(70)
BC	Characterization of tumor heterogeneity	Transcriptomic intratumor heterogeneity exists in BC PDOs	(40)
HER2^+^ BC, TNBC	Mechanisms of proliferation and apoptosis Drug development	Obamatine inhibits BC proliferation and promotes apoptosis by suppressing the PI3K/AKT signaling pathway.	(71)

## 6 Limitations and potential applications

In this review, we primarily discussed the research and application prospects of BC PDO models. PDOs can preserve a part of the TME of the original tissue, cell morphology, tumor heterogeneity, and the patient’s responses to drugs. Moreover, even after several passages, most of their features are preserved. However, the current PDO models remain immature and imperfect. One limitation of the PDO model is that it cannot generalize the entire TME of the patient and lacks a tumor matrix, blood vessels, and immune cells. In response, Neal et al. co-cultured PDOs with other cellular components to reconstruct the TME (60). Furthermore, Davaadelger et al. added fibroblasts to PDOs to investigate the effect of BRCA1 mutations on progesterone in breast cells (72). In addition, extracellular elements and microorganisms may be co-cultured with PDOs in the future (73, 74).

Organoids can be established from patient-derived tumor tissues and can be efficiently expanded and passaged for further drug screening. This makes PDO a valuable model for translating into practical applications and tailoring individualized cancer treatments. One of the most promising applications of PDOs is their ability to predict a patient’s drug response, possibly guiding individualized cancer treatment. For example, Chen et al. successfully guided the individualized treatment of six patients with BC via the drug sensitivity experiments of their PDOs; all patients achieved good efficacy (38). However, PDOs cannot predict the drug responses of the patient’s whole body.

In contrast, organoids include microfluidic systems with advanced 3D bioprinting technologies as the new technology. By employing various bioprinting techniques (such as extrusion, inkjet, photocuring, etc.), the use of bioinks, such as natural or synthetic polymers, cells, or biomolecules, creates complex and anatomically accurate structures and makes each organoid more uniform in volume, which can simulate the biophysical properties of tumors more realistically (75, 76). For example, Heinrich et al. used 3D printing technology to build mini-brain models of glioblastoma and macrophages to study cell interactions and tested the therapies targeting this interaction (77). In addition, the fluid of the microfluidic device can flow and connect to organoids of different tissue origins, further simulating the exchange of nutrients and substances and the formation of vascular networks between tumors. Therefore, the behavior of tumor metastasis and spread can be better simulated (78, 79). Skardal et al. established a “metastasis-on-a-chip” system and simulated the liver metastasis of colon cancer (80). In BC, Swaminathan et al. used bioprinting to directly print 3D spheres of breast epithelial cells and co-culture them with vascular endothelial cells, thereby successfully creating tissue models that could be used for drug efficacy studies (81). However, the accuracy of 3D printing, the cell survival rate, and how to accurately replicate the heterogeneity of the tumor in the model requires further research and validation.

Finally, organoids exhibit considerable potential in regenerative medicine. In addition to being derived from a patient’s tumor tissues, PDOs can also be developed from a patient’s stem cells. Dai et al. noted that breast organoids can be established from human-induced pluripotent stem cells and can differentiate into breast components in immunodeficient mice to further regenerate into breast-like structures. In the future, the issue of BC cell repair and regeneration may be solved after resection (82).

In general, as a new clinical model, PDOs are not only important to study BC mechanisms but also to screen drugs and guide treatment regimens. In the future, research directions can focus on updating breast organoid culture techniques and how to simulate the TME more comprehensively. In conclusion, PDO technology will continue to be an important tool in BC research and clinical drug screening.

## Author contributions

YS: Writing – original draft. ZG: Writing – review & editing. GC: Writing – original draft. YN: Writing – original draft. CZ: Writing – review & editing. WL: Writing – review & editing. JL: Writing – review & editing.
